# Percutaneous Endoscopic Gastrostomy in the 21st Century—An Overview of 1415 Consecutive Dysphagic Adult Patients

**DOI:** 10.3390/nu17050747

**Published:** 2025-02-20

**Authors:** Ivo Mendes, Francisco Vara-Luiz, Carolina Palma, Gonçalo Nunes, Maria João Lima, Cátia Oliveira, Marta Brito, Ana Paula Santos, Carla Adriana Santos, Tânia Meira, Paulo Mascarenhas, Jorge Fonseca

**Affiliations:** 1 GENE—Artificial Feeding Team, Gastroenterology Department, Hospital Garcia de Orta, 2805-267 Almada, Portugal; 2 Egas Moniz Center for Interdisciplinary Research (CiiEM), Egas Moniz School of Health & Science, 2829-511 Almada, Portugal

**Keywords:** percutaneous endoscopic gastrostomy, PEG, dysphagia, enteral nutrition, tube feeding, survival

## Abstract

**Background/Objectives:** Percutaneous endoscopic gastrostomy (PEG) is recommended for long-term enteral nutrition in dysphagic patients. This study aims to characterize conditions motivating PEG, assess nutritional status on the gastrostomy day, evaluate survival and search for survival predictors. **Methods:** Retrospective study of adult patients who underwent PEG in a tertiary hospital from 2001 to 2023. Data collected included demographics, underlying disorders, nutritional status (anthropometry/laboratory evaluation) on the day of PEG and survival recorded until death or December 2023. Multivariable analysis was performed with Cox regression to search for survival predictors. **Results**: A total of 1415 patients were included (61.8% males, mean age 66.9 years); 66.4% presented a neurological disorder and 31.3% head and neck or esophageal cancers (HNC/EC). The mean BMI was 20.9 kg/m^2^, with 49.8% underweight. Albumin, transferrin and total cholesterol were low at 43.2%, 62.2% and 50%, respectively. Median overall survival was 11.1 months; 14.1% of deaths occurred within 4 weeks. HNC/EC patients showed lower survival than neurological patients. Potentially regressive neurological conditions presented longer survival than progressive ones. Predictors of increased survival included female gender, younger age, higher albumin and higher BMI. The protective effect of BMI and albumin was more pronounced in males than in females. **Conclusions:** Neurological disorders were the most frequent underlying conditions. Nearly half of the patients displayed malnutrition before PEG feeding. Although PEG-fed patients displayed a considerable median survival time, some died early without benefit from PEG. Patients with potentially regressive neurological conditions presented better outcomes. Female gender, younger age, higher albumin and higher BMI were associated with longer survival.

## 1. Introduction

Dysphagia is defined as a subjective sensation of swallowing difficulty. Multiple conditions can lead to its onset, including structural or functional oropharyngeal and esophageal abnormalities, establishing dysphagia as one of the most frequently reported digestive disorders [1]. A significant consequence of dysphagia is malnutrition, as impaired swallowing can lead to inadequate nutritional intake, weight loss and nutrient deficiencies [2,3]. In fact, it is estimated that 39.2% of dysphagic patients are at risk for malnutrition, and 13.6% of individuals at risk for malnutrition have dysphagia [4]. Additionally, pulmonary aspiration is a common complication in dysphagic patients, further complicating nutritional management and increasing the risk of respiratory infections. Early recognition and prompt assessment are crucial to ensure appropriate therapy, and nutritional intervention is mandatory, frequently using enteral tube feeding.

Percutaneous endoscopic gastrostomy (PEG) is widely used to provide access to long-term enteral nutritional support, as well as fluid and medication administration, in dysphagic patients with prolonged insufficient oral intake [5]. In selected dysphagic patients, PEG feeding can reduce the risk for aspiration, reduce distress and improve the quality of life of both patients and their caregivers while being a cost-effective option to provide nutritional support and thus prevent malnutrition. Although PEG tube placement is considered a safe and minimally invasive procedure, and tube feeding is usually well tolerated, complications may still occur [6]. Nevertheless, they are usually minor (mostly infections and related to dysfunction of the enteral access tract), with major complications reported in fewer than 2% of cases [7].

A vast heterogeneous group of underlying conditions can lead to the decision to place a gastrostomy tube. The two main groups of underlying conditions needing long-term enteral feeding are neurological disorders (such as post-stroke dysphagia and amyotrophic lateral sclerosis) and cancers of the upper air–digestive tract (including head and neck malignancies and esophageal cancer) [8,9]. All of them have in common compromised deglutition (dysphagia) due to neuromuscular dysfunction or structural obstruction/compression.

Decision-making for gastrostomy placement requires careful consideration on a case-by-case basis, with the underlying condition, clinical status and prognosis taken into account. This process should include setting realistic goals in collaboration with patients and their families/caregivers, clearly explaining the risks and expected benefits, and addressing their beliefs and preferences [10,11]. As a rule, PEG feeding should be considered when inadequate nutritional intake is expected for a period exceeding a few weeks, with most centers accepting a threshold of more than 3–4 weeks [5,8,10].

Thus, estimating prognosis is a critical factor in the decision-making of PEG tube placement. However, it remains challenging and heavily reliant on clinical judgement, given the lack of universally accepted prognostic models [12]. Although there has been increasing interest in improving patient selection for gastrostomy procedures, literature evidence is scarce and often inconsistent regarding predictors of survival [13,14]. This inconsistency imposes difficulties on the ability of clinicians to make evidence-based decisions. Recognizing this gap, our team has worked on developing outcome prediction tools to assist in outcome estimation tailored to general PEG candidates [12] and for head and neck cancer patients [15,16]. Despite these efforts, decision-making still relies on clinical judgement. This underscores the need for further research to refine patient selection criteria, improve risk stratification and enhance prognostic accuracy in PEG candidates.

Factors such as advanced age and low body mass index (BMI) have been frequently associated with increased mortality risk in several studies [17,18,19,20]. In addition to demographic and anthropometric factors, laboratory biomarkers frequently used to monitor nutritional status in PEG patients have been studied as potential survival predictors. Among these, serum albumin, transferrin and total cholesterol have emerged as potential prognostic indicators, although they are influenced by inflammatory and other disease-related factors [21]. Hypoalbuminemia, in particular, has been most consistently reported as a predictor of early mortality [22,23,24,25]. Other biomarkers, including C-reactive protein and serum sodium, have also been suggested as potential survival predictors in some studies [24,25,26].

In order to investigate factors influencing patient survival, we share insights from our experience with one of the largest series of PEG-fed patients worldwide, focusing on clinically relevant predictors during an extended timeline. We outline the vast experience with PEG feeding of our multidisciplinary nutrition support team (GENE), comprising physicians (gastroenterologists and digestive surgeons), dietitians, pharmacists and nurses. The main goals of this study were to assess the different clinical features that led to the gastrostomy procedure; to evaluate nutritional status on the day of the gastrostomy procedure using anthropometric and laboratory data; and to assess patient survival and identify survival predictors.

## 2. Materials and Methods

### 2.1. Study Design

Single-center, observational and retrospective study performed in a tertiary hospital.

### 2.2. Patients

We studied consecutive patients who underwent percutaneous endoscopic gastrostomy for nutritional support from 1 January 2001 to 31 December 2023. Patients under 18 years of age were excluded. All data are part of the routine evaluation of patients referred to PEG and were collected from the GENE clinical files.

### 2.3. Underlying Conditions

The underlying conditions leading to PEG were categorized into four different groups:

1. Neurological disorders with a progressive course (such as neurodegenerative diseases);
2. Neurological disorders with a potentially regressive course (conditions resulting from sudden aggression, such as stroke or head trauma, where patients tend to improve over time);
3. Head and neck cancer/esophageal cancer (HNC/EC);
4. Other conditions that do not fit in the three previous groups.

### 2.4. Clinical Outcome

Survival was expressed in months, from the date of the PEG procedure to the date of death or until 31 December 2023. Patients alive at the end of the follow-up period were considered censored in the survival analysis. Patients who survived less than four weeks were categorized as short survivors, and patients who survived four weeks or more were categorized as adequate survivors.

### 2.5. Anthropometric Evaluation

Body mass index (BMI) was obtained in most patients using the equation weight/height^2^. In cases where weight measurement was not feasible due to the patient’s inability to stand up, BMI was estimated using the mid-upper-arm circumference (MUAC) and regression equations described by Powell-Tuck and Hennessy [27]. This method is proven to provide a reliable BMI estimation in PEG-feeding patients [28]. MUAC was measured in centimeters using a flexible measuring tape wrapped around the upper arm, halfway between the olecranon and the acromion process. Patients under 65 years old were categorized according to their BMIs as underweight (BMI < 18.5 kg/m^2^), normal (BMI 18.5–24.9 kg/m^2^), overweight (BMI 25–29.9 kg/m^2^) or obese (BMI ≥ 30 kg/m^2^). Patients aged 65 years or older were categorized according to their BMIs as underweight (BMI < 22 kg/m^2^), normal (BMI 22–26.9 kg/m^2^), overweight (BMI 27–29.9 kg/m^2^) or obese (BMI ≥ 30 kg/m^2^).

### 2.6. Laboratory Evaluation

A blood sample was obtained on the day of the gastrostomy procedure, assessing serum levels of albumin, transferrin and total cholesterol. Patients were categorized according to local laboratory reference values as having low albumin (<3.5 g/dL) or normal albumin (≥3.5 g/dL); low transferrin (<200 mg/dL) or normal transferrin (≥200 mg/dL); and low total cholesterol (<160 mg/dL) or normal total cholesterol (≥160 mg/dL).

### 2.7. Statistics

Statistical analysis was performed using the Statistical Package for Social Sciences (IBM SPSS^®^ Statistics, version 29.0). Continuous variables were expressed as means and standard deviations or medians and interquartile ranges. Categorical variables were expressed as total and relative frequencies.

Survival was plotted using the Kaplan–Meier method, and differences between groups were determined with a Log-rank test. To evaluate the correlation between age, BMI, albumin, transferrin and total cholesterol with survival, Spearman’s correlation was used. To compare short survivors and adequate survivors, we used the independent samples *t*-test after assessing normality and homogeneity of the variance with the Kolmogorov–Smirnov and Levene tests, respectively.

Multivariable survival analysis was conducted using a Cox proportional hazards regression analysis to assess the effects of gender, age, BMI, albumin, transferrin, total cholesterol and group of underlying condition on survival after exploring for interactions between predictors.

All inferential tests were performed at a 5% level of statistical significance.

## 3. Results

### 3.1. Patients

From the total number of patients who underwent PEG, patients under 18 years old were excluded. A total of 1415 adult patients were included: 874 males (61.8%) and 541 females (38.2%), aged between 18 and 100 years old (mean 66.9 ± 15.1 years); 833 patients (58.9%) were ≥ 65 years old, and 582 (41.1%) were < 65 years old.

### 3.2. Underlying Conditions

**Figure 1:**
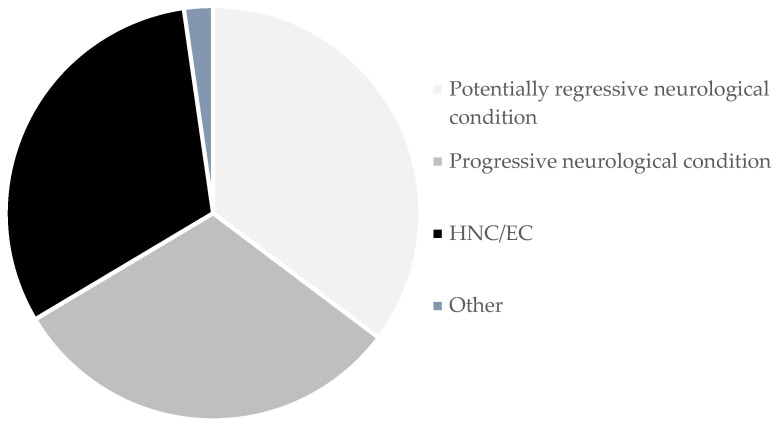
Groups of underlying conditions motivating PEG.

A total of 940 patients (66.4%) presented neurological disorders, 443 patients (31.3%) presented head and neck cancer (HNC) or esophageal cancer (EC) and 32 patients (2.3%) underwent PEG for other conditions (Figure 1). Post-stroke dysphagia was the most common neurological condition, followed by dementia and amyotrophic lateral sclerosis. Laryngeal and pharyngeal cancers were the most common disorders in the HNC/EC group.

Regarding patients having a neurological condition, 499 patients (35.3% of all patients) presented a condition with a potentially regressive course. This included conditions like post-stroke dysphagia, traumatic brain injury and hypoxic-ischemic encephalopathy. A total of 441 patients (31.2%) presented a condition with a progressive course. This included disorders like dementia, amyotrophic lateral sclerosis and parkinsonian syndromes.

More detailed information on the underlying conditions and their distribution between genders is shown in Table 1.

### 3.3. Anthropometric and Laboratory Evaluation

BMI was obtained from 1251 patients, of which 261 were estimated using the Powell-Tuck and Hennessy regression equations. The mean BMI value was 20.9 ± 4.3 kg/m^2^. A total of 623 patients (49.8%) were underweight, 494 patients (39.5%) presented a normal weight, 82 patients (6.6%) were overweight and 52 patients (4.2%) were obese. Blood levels of albumin, transferrin and total cholesterol were obtained from 1297, 1253 and 1243 patients, respectively. Their mean values were 3.5 ± 0.7 g/dL, 186.0 ± 48.7 mg/dL and 163.9 ± 46.9 mg/dL, respectively. A total of 560 patients (43.2%) presented low albumin, 779 (62.2%) presented low transferrin and 622 (50%) presented low total cholesterol. Table 2 summarizes the absolute and relative frequencies of BMI and serum biomarkers assessed in this study.

### 3.4. Clinical Outcome

The estimated median overall survival time was 11.1 [IQR 3.1–42.3] months.

**Figure 2:**
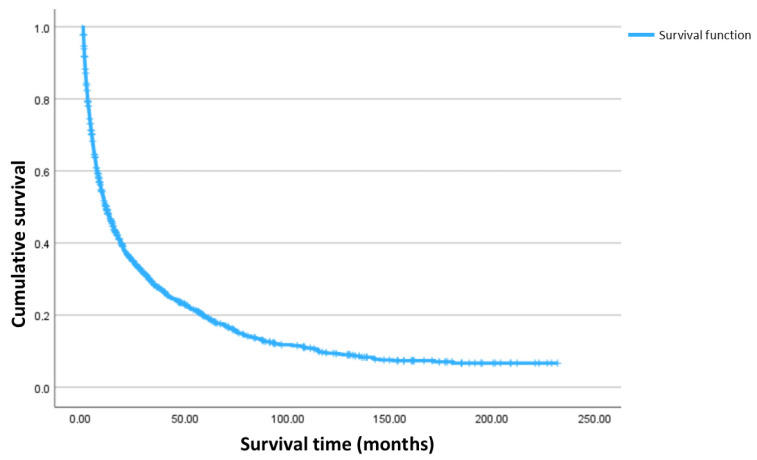
Kaplan–Meier curve of cumulative overall survival.

1-, 3- and 5-year survival rates were 48.2%, 27.8% and 19.6%, respectively. The Kaplan–Meier curve of cumulative overall survival is presented in Figure 2.

Patients having a neurological condition with a potentially regressive course presented a higher survival time when compared to patients having a neurological condition with a progressive course (16.6 vs. 13.3 months, *p* = 0.004). HNC/EC patients presented a significantly lower survival (6.6 months) when compared to both of these groups (*p* < 0.001) (Table 3).

Male patients presented a lower survival time compared to female patients (8.2 vs. 16.0 months, *p* < 0.001) (Table 4).

**Figure 3:**
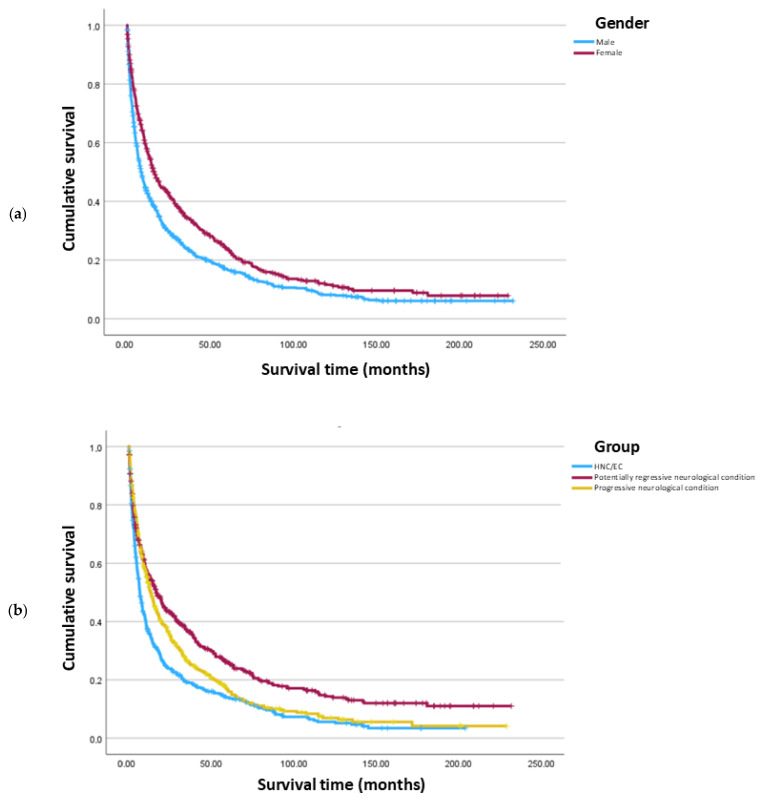
Kaplan–Meier curves of cumulative survival comparing (**a**) gender; and (**b**) group of underlying conditions.

Kaplan–Meier curves of cumulative survival comparing groups of underlying conditions and genders are presented in Figure 3.

Data from Spearman’s correlation demonstrated weak but statistically significant associations between survival and age, BMI, albumin, transferrin and total cholesterol (Table 5). Age showed a negative correlation with survival (r = −0.17, *p* < 0.001), indicating that older patients tended to have shorter survival times. In contrast, BMI, albumin, transferrin and total cholesterol were positively correlated with survival, though the strength of these associations remained weak (BMI: r = 0.12, *p* < 0.001; albumin: r = 0.24, *p* < 0.001; transferrin: r = 0.23, *p* < 0.001; total cholesterol: r = 0.13, *p* < 0.001).

A total of 251 patients were alive at the time of data collection (31 December 2023).

Of the 1164 dead patients, 164 (14.1%) survived less than 1 month (short survivors). This subset of patients exhibited distinct clinical and laboratory differences compared to adequate survivors (Table 6). They were significantly older (70.9 vs. 67.8 years, *p* = 0.008), suggesting that advanced age may be associated with poorer short-term survival. Laboratory markers also differed between the two groups. Short survivors had significantly lower mean albumin (3.1 vs. 3.5 g/dL, *p* < 0.001), transferrin (156.6 vs. 186.6 mg/dL, *p* < 0.001) and total cholesterol levels (151.2 vs. 164.0 mg/dL, *p* = 0.003), indicating a possibly more pronounced state of malnutrition. Although BMI was lower in short survivors (20.4 vs. 20.8 kg/m^2^), this difference did not reach statistical significance (*p* = 0.331).

Survival outcomes were evaluated using a Cox proportional hazards regression analysis after exploring for significant interactions between predictors. The overall model was statistically significant (Waldχ^2^ = 207.32, *p* < 0.001), indicating that the covariates collectively influenced survival outcomes. Results are presented in Table 7. Key findings are listed below:

- Being female is associated with a 60.4% lower risk of death, indicating a significantly reduced hazard compared to males.
- Older age is associated with an increased hazard. Each additional year increases the risk of death by 2.0%.
- Compared to those with a progressive neurological condition, patients with potentially regressive neurological conditions have a decreased hazard, with a 20.9% lower risk of death, while patients with HNC/EC have an increased hazard, with a 39.1% higher risk of death. Patients in the HNC/EC group have an increased hazard compared to patients with potentially regressive neurological conditions, with 75.8% higher risk of death. Patients with other conditions do not differ significantly from the neurological and HNC/EC groups.
- Higher BMI is associated with a reduced hazard. Each unit increase in BMI decreases the risk of death by 3.7%.
- Higher albumin levels are associated with a decreased hazard. Each unit increase in albumin decreases the risk of death by 31.3%.
- A significant interaction between gender, BMI and albumin was observed, indicating that the combined effect of BMI and albumin on survival varies by gender. Specifically, for every one-unit increase in the product of albumin and BMI, the risk of death is approximately 0.8% higher in females when compared to males. This suggests that higher BMI and albumin levels provide greater protection against mortality in males than in females (although they remain protective in females, their effect is slightly attenuated).
- Transferrin and total cholesterol were not significantly associated with survival (*p* > 0.05).

## 4. Discussion

Our study offers a comprehensive analysis of PEG-fed patients over a 23-year period, exploring survival outcomes and factors influencing prognosis. It included 1415 consecutive adult patients who underwent PEG, placing our series as one of the largest series of gastrostomized adult patients worldwide.

Underlying conditions that led to PEG were multiple. Most patients presented a neurological disease (66.4%), and a significant proportion of patients presented head and neck cancer or esophageal cancers (31.3%), leaving only a small percentage of patients (2.3%) with conditions impossible to categorize in one of these two groups. In fact, this is matched with reported literature on underlying conditions leading to PEG [8,9,29].

Among neurological disorders, post-stroke dysphagia was the most frequent. This is primarily explained by the high incidence of stroke, which is one of the leading causes of morbidity in adults, with dysphagia being one of its most disabling sequelae [30,31]. In fact, dysphagia is very frequent in the early phases of stroke. Besides swallowing therapy and dietetic advice with changes in food texture, those with severe dysphagia may need tube feeding with a nasogastric tube or with PEG if enteral feeding is likely necessary for a longer period of time (more than 3–4 weeks) [32]. Nevertheless, only a small percentage of post-stroke dysphagia patients ultimately require PEG tube placement, as the majority recover within the first weeks [33,34].

Dementia was the second most common neurological condition. Malnutrition in this setting arises from a complex interplay of a wide range of factors, including anorexia, food refusal, hypothalamic dysregulation of olfactory and taste functions, feeding apraxia, dysphagia and increased energy expenditure associated with hyperactivity and psychomotor symptoms. Tube feeding in patients with dementia is controversial and frequently raises moral and ethical issues, with conflicting literature results regarding benefits. Nevertheless, it should not be viewed as an absolute contraindication for PEG feeding, and patients should be selected on a case-by-case basis [29,35]. In fact, only patients with moderate-to-severe dementia presenting dysphagia and set in an environment with strong emotional bonds from family and caregivers were considered for gastrostomy. Although dementia patients who underwent PEG were restricted, the high prevalence of dementia (mostly Alzheimer’s and vascular dementia) is the main reason for being the second leading neurological condition.

Amyotrophic lateral sclerosis (ALS) was the third leading neurological cause. In these patients, gastrostomy was discussed regularly at an early stage of the disease, as a gastrostomy procedure before the onset of significant respiratory dysfunction and significant weight loss is associated with lower complication rates and better outcomes [36,37]. In ALS patients, some studies showed a moderate survival advantage for those on PEG nutritional support [38,39]. Although it is a much less frequent disorder when compared to stroke and dementia, almost all ALS patients need PEG feeding in the course of the disease since dysphagia is virtually inevitable [40].

A significant proportion of patients suffered from HNC/EC, with laryngeal and pharyngeal cancers being the most frequent. In fact, these are the cancer types that are more frequently associated with malnutrition, reaching up to 40–60% [41,42]. It is not only a consequence of mechanical obstruction leading to dysphagia and odynophagia but also from dysphagia induced by therapy (surgery, chemotherapy, radiotherapy) and its complications (like mucositis, nausea, dysgeusia and xerostomia) [16,41,43]. In the majority of these patients, especially those undergoing therapy with curative intent, the direct percutaneous introducer (“push”) technique with gastropexy was preferred for PEG tube placement, as it minimizes the risk of tumor seeding at the gastrostomy site. [44,45,46]. Timing for the gastrostomy procedure was always discussed in a multidisciplinary setting. Prophylactic tube placement was mostly preferred in patients undergoing chemoradiation therapy with risk factors for prolonged tube feeding like pretreatment weight loss, advanced age and nasopharyngeal/hypopharyngeal cancer [42].

Although the distribution between genders in the neurological group was almost equal (49.6% males and 50.4% females), the majority of patients in the HNC/EC group were male (87.6% males and 12.4% females). This may be explained by a higher prevalence of risk factors for these cancers (like smoking and alcohol consumption) in males.

Malnutrition was common at the moment of the endoscopic gastrostomy procedure, signaled by a low BMI in almost 50% of patients. Laboratory values of albumin, transferrin and total cholesterol were also low in 43.2%, 62.2% and 50% of patients, respectively. Although these biomarkers are dependent on several non-nutritional influences, they reinforce patients’ malnutrition at the time of the PEG procedure. These findings highlight that referral for PEG procedure often occurs late in the course of the underlying disease, with malnutrition already well established. This delay in addressing nutritional risk often leads to a more pronounced catabolic state, increasing the risk of the procedure and leading to less favorable overall outcomes. Implementing routine nutritional screening and assessment with standardized criteria for referral to nutrition support teams is essential to prioritize prevention and early intervention over reactive measures in malnutrition. Ultimately, shifting the paradigm to earlier referral could decrease complications related to severe malnutrition.

Patients achieved a considerable overall median survival of 11.1 months. However, 14.1% of the deceased patients presented a survival lower than 1 month. This suggests that patient selection was inadequate in those patients, in line with findings from other previous studies [29,47,48]. These short survivors presented lower mean values of albumin, transferrin and total cholesterol and higher mean age when compared to adequate survivors. BMI was lower in the short survivors, although not reaching statistical significance. These clinical and laboratory features should be considered as signaling a high risk of early death and impose a special consideration before choosing to perform a gastrostomy.

The multivariable analysis demonstrated that female gender, a younger age, a higher BMI and higher albumin levels were associated with better survival, while transferrin and total cholesterol levels were not statistically significant. A significant interaction between gender, BMI and albumin was found. It indicated that while albumin and BMI each have their own effects on patient outcomes, their combined impact is not uniform across genders, with higher BMI and albumin levels offering more protection against mortality in males than in females. Although a hazard ratio of 1.008 per unit increase may seem modest, when considering larger variations in BMI and albumin, these effects can accumulate and become clinically meaningful. Regarding underlying conditions, patients with HNC/EC demonstrated a significantly lower survival when compared to those with neurological conditions. Further subdividing the neurological group according to disease course unveiled significant differences, with patients with progressive neurological disorders having a lower survival when compared to patients with potentially regressive neurological disorders. The lack of significant survival differences among patients with conditions outside the neurological and HNC/EC groups (*p* > 0.05) confirms that these patients represent a more heterogeneous group where prognosis varies widely (as reflected by a higher standard deviation value), potentially diluting survival trends. This underscores the importance of individualized patient evaluation in such cases. Although the existing literature is limited, our findings align with previous studies since younger age, higher BMI and normal serum albumin are commonly reported as survival predictors. However, prior results remain conflicting, particularly regarding gender and underlying disorders [17,18,19,20,22,23,24,25,29,48].

Our study has several notable strengths, including being a real-life large sample study with an extended follow-up period. However, some limitations must be acknowledged. Firstly, as a retrospective study, it is subject to inherent biases, such as information bias. In fact, data were collected from existing records, which were sometimes incomplete (e.g., missing BMI and laboratory biomarkers) and prone to inaccuracies. Additionally, as discussed previously, indication and timing for PEG tube placement in some specific disorders are not consensual across institutions. Thus, as this is a single-center study, institutional practices may influence results. Furthermore, biomarkers such as C-reactive protein and sodium levels, which some studies have identified as potential predictors of survival, were not evaluated in this study due to the inconsistency of the available records.

To minimize these limitations in future research, prospective and multicenter studies considering a broader range of potential survival predictors should be conducted. This would allow for the collection of more comprehensive data and provide better control over potential confounding variables, leading to more robust and generalizable results.

We have witnessed and experienced the development of PEG feeding in the 21st century and have accumulated substantial knowledge in the management of PEG patients. However, while estimating prognosis is a critical factor in the decision of PEG tube placement, it still remains tightly dependent on clinical judgment, which can lead to suboptimal patient selection. The findings highlight the complexity of patient profiles and underline significant predictors of survival, contributing to valuable insights into improving decision-making in PEG tube placement. Our results reinforce age, gender, underlying disorders, BMI and albumin levels as predictors for survival in PEG patients. These factors should be taken into account in a multidisciplinary approach to guide decision-making, focusing on earlier nutritional intervention and improved patient selection to ensure that PEG is offered to patients who are most likely to benefit while avoiding unnecessary interventions in those who do not. We acknowledge the potential advantage of subdividing neurological conditions by their clinical course (as progressive or potentially regressive) since this unveiled significant differences in survival analysis. This subclassification is clinically relevant and can offer a more tailored and refined approach to prognostic assessment. However, future studies are warranted to validate these findings and to integrate them into prognostic models that could improve patient selection.

## 5. Conclusions

The underlying conditions leading to PEG tube placement in long-term dysphagic patients were diverse, with neurological disorders being the most frequent, followed by HNC/EC. Almost 50% of patients were malnourished at the time of the gastrostomy procedure, reflected by a low BMI as well as low laboratory values of biomarkers like albumin, transferrin and total cholesterol. Patients presented a considerable median survival after the PEG procedure. Nevertheless, some survived less than 1 month, suggesting that patient selection must improve.

Multivariable analysis revealed that female gender, younger age, higher albumin levels and higher BMI are predictors of longer survival. The protective effect of higher BMI and albumin levels was more pronounced in males than in females. HNC/EC patients presented a significantly lower median survival compared to neurological patients. Furthermore, dividing neurological disorders according to disease course revealed a better outcome for patients with potentially regressive conditions when compared to those with progressive conditions. Given the results, the authors underscore the need for earlier nutritional intervention and the consideration of these survival predictors to improve decision-making for PEG candidates.

The findings emphasize the complex interplay of clinical, demographic and nutritional factors influencing survival after PEG tube placement. By integrating these predictors of survival into clinical practice, patient selection can be tailored in order to optimize outcomes.

## Figures and Tables

**Table 1:** Underlying conditions motivating PEG and distribution between genders.

	Total: *n* (%)	Male: *n* (%)	Female: *n* (%)
Potentially regressive neurological condition	499 (35.3)	273 (54.7)	226 (45.3)
Post-stroke dysphagia	361 (25.5)	177 (49.0)	184 (51.0)
Traumatic brain injury	78 (5.5)	55 (70.5)	23 (29.5)
Hypoxic-ischemic encephalopathy	33 (2.3)	23 (69.7)	10 (30.3)
Cerebral palsy	10 (0.7)	6 (60.0)	4 (40.0)
Other	17 (1.2)	12 (70.6)	5 (29.4)
Progressive neurological condition	441 (31.2)	193 (43.8)	248 (56.2)
Dementia	155 (11.0)	61 (39.4)	94 (60.6)
Amyotrophic lateral sclerosis	153 (10.8)	76 (49.7)	77 (50.3)
Parkinsonian syndromes	39 (2.8)	18 (46.2)	21 (53.8)
Cerebral neoplasm	39 (2.8)	15 (38.5)	24 (61.5)
Huntington’s disease	13 (0.9)	5 (38.5)	8 (61.5)
Oculopharyngeal muscular dystrophy	11 (0.8)	5 (45.5)	6 (54.5)
Other	31 (2.2)	13 (41.9)	18 (58.1)
Head and neck cancer/Esophageal cancer	443 (31.3)	388 (87.6)	55 (12.5)
Laryngeal cancer	135 (9.5)	127 (94.1)	8 (5.9)
Pharyngeal cancer	132 (9.3)	118 (89.4)	14 (10.6)
Oral cavity cancer	98 (6.9)	85 (86.7)	13 (13.3)
Esophageal cancer	54 (3.8)	46 (85.2)	8 (14.8)
Other	24 (1.7)	12 (50.0)	12 (50.0)
Other conditions	32 (2.3)	20 (62.5)	12 (37.5)

**Table 2:** BMI and serum biomarkers.

	Total: *n* (%)
BMI (*n* = 1251)	
Underweight	623 (49.8)
Normal	494 (39.5)
Overweight	82 (6.6)
Obesity	52 (4.2)
Albumin (*n* = 1297)	
Low (<3.5 g/dL)	560 (43.2)
Normal (≥3.5 g/dL)	737 (56.8)
Transferrin (*n* = 1253)	
Low (<200 mg/dL)	779 (62.2)
Normal (≥200 mg/dL)	474 (37.8)
Total cholesterol (*n* = 1243)	
Low (<160 mg/dL)	622 (50.0)
Normal (≥160 mg/dL)	621 (50.0)

**Table 3:** Survival of patients comparing groups of underlying conditions.

	Survival		95% CI		Log-Rank (Mantel–Cox)	
	Median (Months)	Std. Error	Lower Bound	Upper Bound	Chi-Square	*p*-Value
Neurological regressive	16.6	1.9	12.9	20.2	-	-
Neurological progressive	13.3	1.1	11.1	15.5	8.50	0.004
HNC/EC	6.6	0.6	5.4	7.7	37.91	<0.001
Others	6.4	13.6	0.0	33.1	0.07	0.793
Neurological progressive	13.3	1.1	11.1	15.5	-	-
HNC/EC	6.6	0.6	5.4	7.7	13.23	<0.001
Others	6.4	13.6	0.0	33.1	2.15	0.143
HNC/EC	6.6	0.6	5.4	7.7	-	-
Others	6.4	13.6	0.0	33.1	5.14	0.023

**Table 4:** Survival of patients across genders.

	Survival		95% CI		Log-Rank (Mantel–Cox)	
	Median (Months)	Std. Error	Lower Bound	Upper Bound	Chi-Square	*p*-Value
Female	16.0	1.44	13.14	18.80	-	-
Male	8.2	0.74	6.79	9.67	20.84	<0.001

**Table 5:** Spearman’s correlation between survival and age, BMI, albumin, transferrin and total cholesterol.

	Correlation Coefficient (r)	*p*-Value
Age	−0.17	<0.001
BMI	0.12	<0.001
Albumin	0.24	<0.001
Transferrin	0.23	<0.001
Total cholesterol	0.13	<0.001

**Table 6:** Comparison of age, BMI, albumin, transferrin and total cholesterol between short and adequate survivors.

	Adequate Survivors (mean ± s.d.)	Short Survivors (mean ± s.d.)	*p*-Value
Age	67.8 ± 14.3	70.9 ± 13.6	0.008
BMI	20.8 ± 4.3	20.4 ± 4.6	0.331
Albumin	3.5 ± 0.7	3.1 ± 0.6	<0.001
Transferrin	186.6 ± 48.3	156.6 ± 46.3	<0.001
Total cholesterol	164.0 ± 46.8	151.2 ± 51.0	0.003

**Table 7:** Results from the Cox proportional hazards regression analysis.

	HR	95% CI		*p*-Value
		Lower Bound	Upper Bound	
Gender	0.396	0.245	0.641	<0.001
Age	1.020	1.015	1.025	<0.001
BMI	0.963	0.946	0.981	<0.001
Albumin	0.687	0.619	0.763	<0.001
Group of underlying condition				
Neurological progressive (ref.) vs. Neurological regressive	0.791	0.670	0.935	0.006
Neurological progressive (ref.) vs.HNC/EC	1.391	1.169	1.656	<0.001
Neurological progressive (ref.) vs. Others	0.730	0.451	1.183	0.202
Neurological regressive (ref.) vs. HNC/EC	1.758	1.483	2.084	<0.001
Neurological regressive (ref.) vs. Others	0.923	0.572	1.489	0.743
HNC/EC (ref.) vs. Others	0.525	0.325	0.847	0.080
Gender x BMI x Albumin	1.008	1.002	1.015	0.008

## Data Availability

The data presented in this study are available upon request due to ethical reasons.
